# Somatosensory Gating Is Modulated by Anodal Transcranial Direct Current Stimulation

**DOI:** 10.3389/fnins.2021.651253

**Published:** 2021-09-07

**Authors:** Casandra I. Montoro, Christine Winterholler, Juan L. Terrasa, Pedro Montoya

**Affiliations:** Research Institute of Health Sciences (IUNICS), Balearic Islands Health Research Institute (IdISBa), University of the Balearic Islands (UIB), Palma, Spain

**Keywords:** late positive complex, cortical excitability, sensory gating, somatosensory cortex, transcranial direct current stimulation

## Abstract

**Background:**

Anodal transcranial direct current stimulation (tDCS) of the somatosensory cortex causes cerebral hyperexcitability and a significant enhancement in pain thresholds and tactile spatial acuity. Sensory gating is a brain mechanism to suppress irrelevant incoming inputs, which is elicited by presenting pairs of identical stimuli (S1 and S2) within short time intervals between stimuli (e.g., 500 ms).

**Objectives/Hypothesis:**

The present study addressed the question of whether tDCS could modulate the brain correlates of this inhibitory mechanism.

**Methods:**

Forty-one healthy individuals aged 18–26 years participated in the study and were randomly assigned to tDCS (*n* = 21) or SHAM (*n* = 20). Somatosensory evoked potentials (SEP) elicited by S1 and S2 pneumatic stimuli (duration of 100 ms, ISI 550 ± 50 ms) and applied to the index finger of the dominant hand were recorded before and after tDCS.

**Results:**

Before the intervention, the second tactile stimuli significantly attenuated the amplitudes of P50, N100, and the late positive complex (LPC, mean amplitude in the time window 150–350) compared to the first stimuli. This confirmed that sensory gating is a widespread brain inhibitory mechanism that can affect early- and middle-latency components of SEPs. Furthermore, our data revealed that this response attenuation or sensory gating (computed as S1 minus S2) was improved after tDCS for LPC, while no changes were found in participants who received SHAM.

**Conclusion:**

All these findings suggested that anodal tDCS might modulate brain excitability leading to an enhancement of inhibitory mechanisms elicited in response to repetitive somatosensory stimuli during late stages of information processing.

## Introduction

Transcranial Direct Current Stimulation (tDCS) involves the safe, painless, and non-invasively application of weak electrical current (commonly ≤ 2 mA) to the cerebral cortex (Antal et al., 2017; Lefaucheur et al., 2017; Matsumoto and Ugawa, 2017). tDCS influences excitability by inducing long-lasting and widespread changes in the brain (Lefaucheur, 2008; Paulus, 2011; Moliadze et al., 2012). Indeed, several studies have suggested that tDCS may cause polarity-dependent shifts of the resting membrane potential and, therefore, changes in neuron firing rates (Furubayashi et al., 2008; Zaghi et al., 2010; Stagg and Nitsche, 2011; Zaehle et al., 2011). Furthermore, it seems that anodal tDCS would enhance cortical excitability by depolarizing neurons, while cathodal tDCS would reduce cortical excitability by inducing hyperpolarization (Nitsche and Paulus, 2000, 2001; Nitsche et al., 2008). Accordingly, it has been shown that tDCS can elicit changes in perfusion and functional connectivity of several cerebral areas (Stagg et al., 2013).

Although most tDCS studies have focused on the stimulation effects of primary motor and dorsolateral prefrontal cortices (Nitsche et al., 2003; Fregni et al., 2005, 2008; Antal et al., 2011; Ahn et al., 2017; Lefaucheur et al., 2017; Vanderhasselt et al., 2017; Angius et al., 2019; Shilo and Lavidor, 2019; Chrysikou et al., 2019; Gallucci et al., 2020), there is evidence that tDCS of the somatosensory cortices can also elicit neurophysiological and psychological after-effects on body processing (Antal et al., 2006, 2008; Dieckhöfer et al., 2006; Lenoir et al., 2017; Saito et al., 2019). Previous research has established that anodal tDCS of the somatosensory cortex elicits brain hyperexcitability, significant enhancement of pain thresholds (Matsunaga et al., 2004; Antal et al., 2008; Grundmann et al., 2011; Vaseghi et al., 2015), and tactile spatial acuity (Ragert et al., 2008; Fujimoto et al., 2014, 2016), whereas cathodal tDCS can reduce the responsiveness of the primary somatosensory cortex to painful and non-painful stimuli (Grundmann et al., 2011; Lenoir et al., 2017).

An easy, reliable, and robust tool for examining the excitability of the somatosensory system and psychological variables involved in the processing of bodily information is the recording of somatosensory evoked potentials (SEPs) (Gibson et al., 1994; Lorenz et al., 1996; Montoya et al., 2005). It is known that SEP amplitudes at early latencies (20–200 ms, depending on stimulation modality) appear to originate in the primary somatosensory cortex and are associated with sensory and attentional processing of somatosensory information (Freedman et al., 1987; Näätänen and Picton, 1987), whereas SEP amplitudes at later latencies (200–500 ms) are linked to more complex cognitive processes such as memory and stimulus evaluation (Polich and Herbst, 2000). Previous studies from our lab have further demonstrated that early- and late-latency SEP amplitudes elicited by non-painful tactile stimulation can be modulated by affect (Montoya et al., 2006), the observation of other’s pain and touch (Martínez-Jauand et al., 2012), chronic pain (Montoya et al., 2005), or aging (Terrasa et al., 2018). Moreover, studies examining the modulatory effects of tDCS of the sensorimotor cortex on SEP amplitudes have demonstrated that cathodal tDCS significantly diminished the amplitudes of early SEP components elicited by electrical (Dieckhöfer et al., 2006), laser (Antal et al., 2008), and vibrotactile stimulation (Lenoir et al., 2017), whereas anodal tDCS resulted in long lasting increase in the amplitudes of early SEP components (P22/N30, P25/N33, and N33/P40) elicited by electrical stimulation (Matsunaga et al., 2004). In the present study, we addressed the question of whether anodal tDCS could also influence amplitudes of early and late SEP components elicited by non-painful tactile stimulation.

It is well-known that the repetitive presentation of paired stimuli can provoke the so-called sensory gating phenomenon, which refers to the ability of the central nervous system to inhibit the processing of irrelevant sensory inputs and represents an essential protective mechanism that prevents flooding of the upper brain centers (Freedman et al., 1987; Cromwell et al., 2008). Sensory gating is elicited by presenting pairs of identical stimuli (S1 and S2) within short time intervals between stimuli (e.g., 500 ms), and is usually calculated as the difference in SEP amplitudes (S1 and S2) or the ratio between SEP amplitudes elicited by S2 and S1 stimuli (S2/S1) (Fuerst et al., 2007). It has been argued that the first stimulus of the pair could activate some inhibitory brain pathways that suppress the response to the second stimulus presented a short time later and, hence, lower ratios or larger differences may indicate greater gating or inhibition of irrelevant inputs (Boutros and Belger, 1999). Consistent with previous findings showing that anodal tDCS causes brain hyperexcitability and significant changes in pain sensitivity and somatosensory processing, we hypothesize here that anodal tDCS of the somatosensory cortex would also result in increased sensory gating due to enhancement of cortical excitability.

## Materials and Methods

### Participants

Forty-one university students (31 women and 10 men) aged between 19 and 44 years were enrolled in the study. None of the participants suffered from any psychiatric, neurological or cardiovascular disease, had contraindications for tDCS or a history of drug abuse, or was receiving pharmacological treatment that affected the cardiovascular or central nervous systems. Thirty-six participants were right-handed and five left-handed according to the Edinburgh Handedness Inventory (Oldfield, 1971). All individuals were naive to the experiment, gave written informed consent, and received course credits for their participation.

The study was conducted in accordance with the Declaration of Helsinki (1991) and was approved by the Ethics Committee of the Balearic Islands (protocol IB3681/18PI).

### Psychological Assessment

A clinical psychologist obtained data about clinical symptoms, medication use, sociodemographic, and psychological characteristics. Depression and anxiety were evaluated by using the Spanish versions of the Beck’s Depression Inventory (BDI; Beck et al., 1961) and the State–Trait Anxiety Inventory (STAI; Spielberger et al., 1970). In addition, all participants completed the Spanish version of the Toronto Alexithymia Scale (TAS-20; Bagby et al., 1994). The TAS-20 is a self-report scale that is comprised of 20 items grouped into three subscales: difficulty identifying feelings (seven items), difficulty describing feelings (five items), and externally oriented thinking (eight items). Items are rated using a 5-point Likert scale (whereby 1 = strongly disagree and 5 = strongly agree). The total alexithymia score was also obtained.

### Transcranial Electrical Stimulation

**FIGURE 1:**
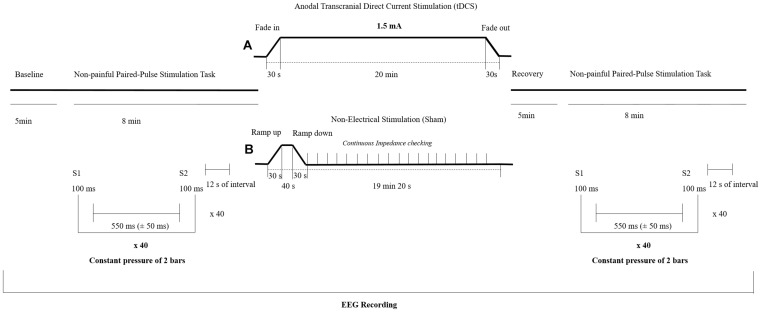
Experimental design of the study. Participants were randomly assigned to either a transcranial direct current stimulation (tDCS) **(A)** or a SHAM group **(B)**. EEG = Electroencephalography.

Transcranial Direct Current Stimulation was delivered by a battery-driven constant-current stimulator (NeuroConn GmbH, Ilmenau, Germany) through two gel conductive-rubber electrodes (diameter: 20 mm; area: 3 cm^2^; thickness of 1 mm). The stimulation electrode (anode) was placed on the scalp over the left (right-handed participants) or right (left-handed participants) somatosensory cortex (CP3 or CP4 electrode location, respectively), while the reference electrode (cathode) was placed over the contralateral supraorbital ridge. Anodal tDCS was applied during 20 min with a current intensity of 1.5 mA and fade-in/fade-out periods of 30 s. The SHAM (non-electrical stimulation) procedure consisted of an initial ramp-up in which the stimulator reached the maximum programmed current (30 s to reach 1.5 mA) followed by a a short stimulatory period (40 s) and a final ramp-down (30 s) involving the current gradually being switched off. The current remained off (i.e. without electrical stimulation) for the rest of the session (19 min 20 s). The impedance was kept under 5 kΩ during the stimulation session. During SHAM session a continuous impedance control was performed in order to detect electrodes slipping off and to show real values at the display (see Figure 1). Participants were instructed to sit comfortably and to rest during all the experimental session. All the parameters used for the electrical stimulation were chosen following the standard guides for design and implementation of the tDCS (Thair et al., 2017). Participants completed a self-report questionnaire after the stimulation to explore the bodily sensations elicited by the intervention (Antal et al., 2017).

### Non-painful Paired-Pulse Stimulation Task

Before and after the tDCS session, participants received tactile paired-pulse stimuli to the forefinger of the dominant hand in order to analyze somatosensory brain processing. The tactile stimulation task consisted of two identical non-painful pneumatic stimuli (S1 and S2) of 100 ms duration. Stimulation was applied with a constant pressure of 2 bars, a randomized inter stimulus interval of 550 ms (±50 ms) and separated by a fixed interval of 12 s. The pneumatic stimulator was used in previous research under the same procedure (Montoya et al., 2006; Terrasa et al., 2018) and consisted of a small membrane attached to the body surface by a plastic clip and fixated with adhesive strips. Forty trials were presented before and 5 min after the application of anodal tDCS or SHAM, respectively, by the usage of a standard software (Presentation version 18.3, Neurobehavioral Systems, Inc., Berkeley, CA, United States)^1^. During this non-painful paired-pulse stimulation task, participants were instructed to rest and attend to the tactile stimuli. The experimental design of the study is shown in the Figure 1.

### Electroencephalography Recording and Data Reduction

Electroencephalography (EEG) was recorded with a commercial amplifier (QuickAmp, Brain Products GmbH, Munich, Germany) with 62 Ag/AgCl electrodes placed according to the 10–10 International System with common average reference, at a sampling rate of 1,000 Hz. A ground electrode was located at position AFz. An electroculogram (EOG) was also recorded by placing one electrode above and one below the left eye. All electrode impedances were kept lower than 10 kΩ.

EEG signals were segmented offline in epochs of 600 ms (–100 to 500 ms relative to the stimulus onset), digitally filtered (high-pass at 0.10 Hz, low-pass at 30 Hz), and baseline corrected (from –100 to 0 ms). Eye movement artifacts were corrected by using the Gratton and Coles algorithm (Gratton et al., 1983). An artifact rejection protocol with the following criteria was applied: maximal allowed voltage step/sampling point = 75 μV, minimal allowed amplitude = –75 μV, maximal allowed amplitude = 75 μV, and maximal allowed absolute difference in the epoch = 75 μV. All participants fulfilled the further analyses inclusion criteria of keeping at least 75% of the epochs free of artifacts for each stimulus. Then, EEG epochs were separately averaged for S1 and S2.

The amplitudes of the following components of these SEPs were determined: P50, N100, and late positive complex (LPC). The peak amplitude of P50 and N100 was calculated by searching the global maxima at Cz within two time windows after stimulus onset: 20–80 ms for P50, and 80–135 ms for N100. The area under the curve for the time window 150–350 ms was computed for LPC amplitudes. All the reported data pre-processing was done by the software Brain Vision Analyzer version 1.05 (Brain Products GmbH, Munich, Germany).

### Procedure

Participants were randomly assigned to either a SHAM (*n* = 20) or an active tDCS (*n* = 21) intervention. Randomization was performed using the order of entry to the study and a previous computer-generated randomization list with blinded stimulation codes for the investigator and the participants. After the participants signed the informed consent form and completed the psychological questionnaires, they were taken to the EEG laboratory, sat comfortably in a recording chamber, and electrodes for EEG recording and tDCS were placed on the scalp. The EEG recording session started with a 5-min eyes-open resting baseline, followed by the non-painful paired-pulse stimulation task as described above. After a short break (less than 2 min), participants received 20 min of brain stimulation (anodal tDCS or SHAM), followed by a short break to complete the Transcranial Electrical Stimulation Sensation Questionnaire (Antal et al., 2017). The EEG recording session ended with a 5-min rest eyes open baseline and a repetition of the paired pulse stimulation task as described above.

All EEG recording sessions started at 9:00 a.m., 12:00 p.m., or 4 p.m. Participants were instructed to refrain from caffeine, alcohol, nicotine, and vigorous exercise at least 2 h before arriving at the laboratory. Furthermore, they were told to have their breakfast (for morning participants) or lunch (for afternoon participants) as soon as possible and refrain from eating immediately before the experiment.

### Statistical Analyses

Group differences in sociodemographic data and self-report questionnaires were analyzed with non-parametric tests (Chi-Square, Mann–Whitney). For statistical analyses of SEP amplitudes, two separated analyses were performed. The first analysis aimed to examine the effects of the tDCS intervention on the SEP amplitudes of the components (P50, N100, and LPC) elicited by S1 and S2. For this purpose, multivariate analyses of variance (MANOVA) for repeated-measures with the factors “group” (tDCS vs. SHAM), “time” (pre vs. post), and “stimulus” (S1 and S2) were computed on a subset of 12 electrodes (F1, F2, F3, F4, C1, C2, C3, C4, P1, P2, P3, and P4). In addition, the electrodes were grouped according to within-subject factors “Region of Interest (ROI)” (frontal, central, and parietal) and “hemisphere” (left vs. right) for testing topographical effects. The second analysis was aimed to examine the effects of the tDCS intervention on the sensory gating scores obtained for the P50, N100, and LPC amplitudes. For this purpose, the sensory gating scores for each SEP component was computed as the difference between the amplitudes elicited by the first and the second stimuli (S1–S2) at nine electrode locations grouped into three ROIs: frontal (F3, Fz, and F4), central (C3, Cz, and C4) and parietal (P3, Pz, and P4). Thus, these MANOVAs were computed with the factors “group” (tDCS vs. SHAM), “time” (pre vs. post), and “ROI” (frontal, central, and parietal). *Post hoc* mean comparisons were used to further explore significant effects. The level of significance was set at *p* ≤ 0.05 (2-tailed). Greenhouse-Geisser adjustments and Bonferroni corrections were applied if necessary.

## Results

Analyses revealed no significant differences on sociodemographic and psychological data between participants receiving tDCS or SHAM (Table 1). After the intervention, there were no significant group differences in treatment beliefs: eight out of 21 subjects in the tDCS and eleven out of 20 subjects in the SHAM group believed that they were receiving real tDCS [χ^2^ (1) = 1.18, *p* = 0.278]. Furthermore, 10 subjects in the tDCS and 10 in the SHAM group reported the perception of sensations located around either the anodal or the cathodal electrodes [χ^2^ (1) = 0.023, *p* = 0.879]. The mean intensity of any perceived sensations during the procedure according to the questionnaire of sensations related to transcranial electrical stimulation (Antal et al., 2017) is displayed in Table 2. No significant group differences on the intensity of perceived sensations were observed.

**TABLE 1:** Psychological and sociodemographic data.

	tDCS *n* = 21	SHAM *n* = 20	χ^2^ or *t*	*p*	η^2^
Age	22.86 ± 6.41	20.95 ± 2.72	1.23	0.226	0.037
Sex	5/16	5/15	0.01	0.929	0.014
BMI	24.55 ± 5.37	24.65 ± 5.61	−0.06	0.951	0.000
BDI	7.52 ± 7.17	8.60 ± 4.28	−0.59	0.561	0.009
STAI-T	11.26 ± 2.45	11.66 ± 2.61	−0.07	0.945	0.000
STAI-S	10.94 ± 2.39	9.14 ± 2.04	−0.27	0.790	0.002
TA	8.66 ± 1.89	11.29 ± 2.53	0.52	0.612	0.007
DIF	4.17 ± 0.91	4.15 ± 0.93	−0.75	0.457	0.014
DDF	4.52 ± 0.99	4.77 ± 1.07	0.47	0.641	0.006
EOT	4.36 ± 0.95	5.40 ± 1.21	1.24	0.223	0.038

*Sex, men/women; BMI, body mass index; BDI, depression; STAI-T, trait-anxiety; STAI-S, State-anxiety; TA, total alexithymia (TAS-20); DDF, difficulty describing feelings (TAS-20); DIF, difficulty identifying feelings (TAS-20); EOT, external oriented thinking (TAS-20).*

**TABLE 2:** Group comparisons on intensity of perceived sensations from the Questionnaire of sensations related to transcranial electrical stimulation (Antal et al., 2017).

	tDCS (*n* = 21)	SHAM (*n* = 20)	*Z*	*p*	η^2^
Itching	1.09 (0.83)	0.90 (0.72)	−0.807	0.420	0.128
Pain	0.29 (0.46)	0.40 (0.68)	−0.295	0.768	0.101
Burning	0.43 (0.87)	0.40 (0.75)	−0.035	0.972	0.018
Warmth/heat	0.38 (0.59)	0.60 (0.82)	−0.833	0.405	0.156
Metallic/iron taste	0.04 (0.22)	0.05 (0.22)	−0.035	0.972	0.006
Fatigue/decreased alertness	1.00 (1.09)	1.10 (0.79)	−0.592	0.574	0.053

*Degree of intensity measured according to the following scale: 0 = none, 1 = mild, 2 = moderate, and 3 = strong (standard deviations in parenthesis). Group differences were tested with non-parametric Mann–Whitney.*

### Effects of Anodal tDCS on SEP Amplitudes Elicited by Non-painful Paired-Pulses

**FIGURE 2:**
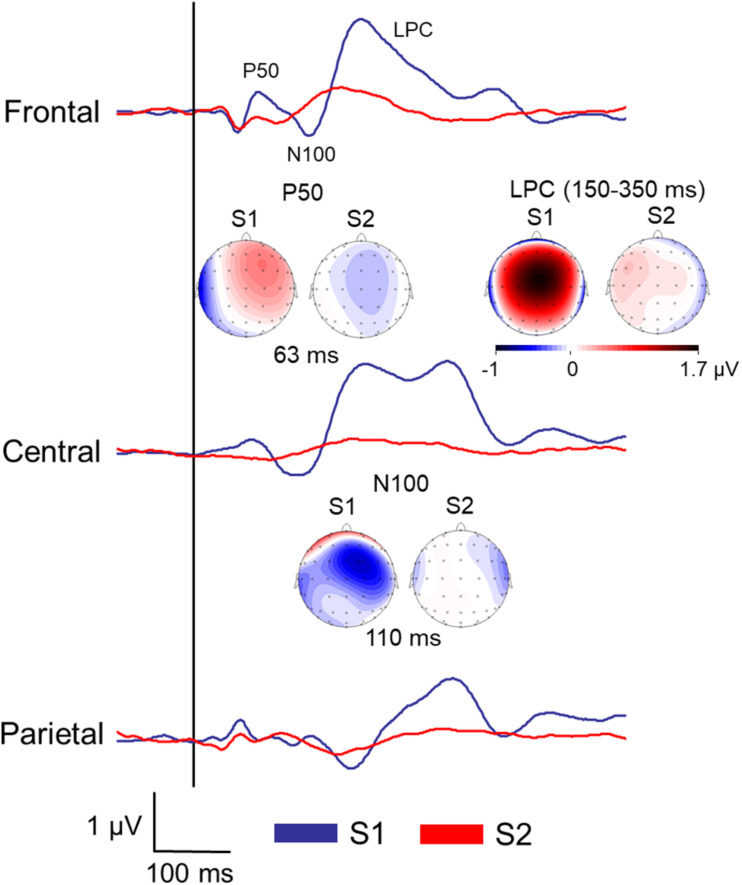
Grand averages of the somatosensory ERPs elicited by the first (S1) and the second stimuli (S2) of all the participants before the brain stimulation (pre) at the three regions of interest (ROI). Topographical maps of the P50 and N100 amplitudes at specific latencies, and late positive complex (LPC) mean amplitude were also included.

Figure 2 displays the grand averages of the SEP elicited by the first (S1) and the second stimuli (S2) before the brain stimulation condition at the three ROIs (frontal, central, and parietal).

#### P50 Amplitudes

As expected, the first MANOVA revealed that overall P50 amplitudes elicited by S1 (0.29 ± 0.08 μV) were greater than those elicited by S2 (−0.04 ± 0.03 μV) [*F*(1,39) = 17.57, *p* ≤ 0.001, η^2^ = 0.310]. In addition, significant effects due to *hemisphere* [*F*(1,39) = 8.28, *p* = 0.006, η^2^ = 0.175] and *hemisphere* × *stimulus* [*F*(1,39) = 9.33, *p* = 0.004, η^2^ = 0.193] indicated that greater P50 amplitudes were elicited by S1 over the right (0.44 ± 0.09 μV) than over the left hemisphere (0.14 ± 0.09 μV; *p* = 0.002), while no hemispheric differences were observed on P50 amplitudes elicited by S2 (*p* = 0.374). Finally, a significant effect due to *ROI* × *stimulus* [*F*(2,38) = 3.73, *p* = 0.047, η^2^ = 0.087] indicated that greater P50 amplitudes were elicited by S1 than by S2 at *frontal* (0.38 ± 0.13 μV for S1 vs. −0.01 ± 0.05 μV for S2; *p* = 0.001) and *central* regions (0.39 ± 0.09 μV for S1 vs. −0.06 ± 0.05 μV for S2; *p* ≤ 0.001), while no differences were observed at parietal ones (*p* = 0.09). No effects due to *time* or to *group* were found at any of the three ROIs.

#### N100 Amplitudes

A significant effect due to *stimulus* was found [*F*(1,39) = 9.05, *p* = 0.005, and η^2^ = 0.188], indicating that S1 elicited greater N100 amplitudes (−0.31 ± 0.06 μV) than S2 (−0.14 ± 0.04 μV). Furthermore, significant effects due to *hemisphere* × *time* [*F*(1,39) = 5.29, *p* = 0.027, and η^2^ = 0.120] and *ROI* × *hemisphere* × *stimulus* [*F*(2,38) = 3.97, *p* = 0.025, and η^2^ = 0.092] were found. However, *post hoc* mean comparisons revealed that there were no hemispheric differences in N100 amplitudes elicited by either S1 or S2, and that there were no differences between N100 amplitudes elicited by S1 and S2 over either the right or left hemisphere at any ROI. No effects due to *time*, *group* or *time* × *group* were found at any of the three ROIs.

#### LPC Amplitudes

A significant effect due to *stimulus* was found [*F*(1,39) = 32.62, *p* ≤ 0.001, and η^2^ = 0.455], indicating that S1 elicited overall greater LPC amplitudes (167.04 ± 25.32 μV × ms) than S2 (10.63 ± 6.55 μV × ms). Significant effects due *hemisphere* × *stimulus* [*F*(1,39) = 4.35, *p* = 0.044, and η^2^ = 0.100] further indicated that S2 elicited higher LPC amplitudes over the left (26.50 ± 8.06 μV × ms) than over the right hemisphere (−5.24 ± 11.66 μV × ms; *p* = 0.043), whereas no hemispheric differences were observed on LPC amplitudes elicited by S1 (*p* = 0.277). Additionally, significant effects due to *ROI* [*F*(2,38) = 7.63, *p* = 0.001, and η^2^ = 0.164], and *ROI* × *stimulus* [*F*(2,38) = 4.64, *p* = 0.013, and η^2^ = 0.106] revealed that LPC amplitudes elicited by S1 were higher than those elicited by S2 at *frontal* (S1: 176.83 ± 31.51 μV × ms; S2: 30.18 ± 10.10 μV × ms; *p* ≤ 0.001), *central* (S1: 245.84 ± 50.65 μV × ms; S2: 9.30 ± 10.30 μV × ms; and *p* ≤ 0.001) and *parietal* electrode locations (S1: 78.45 ± 25.01 μV × ms; S2: −7.59 ± 9.67 μV × ms; and *p* = 0.002). Finally, significant differences due to *ROI* × *time* [*F*(2,38) = 5.38, *p* = 0.019, and η^2^ = 0.121], *hemisphere* × *group* [*F*(1,39) = 5.38, *p* = 0.026, and η^2^ = 0.121], *stimulus* × *time* [*F*(1,39) = 5.65, *p* = 0.022, and η^2^ = 0.127] and *stimulus* × *time* × *group* [*F*(1,39) = 4.82, *p* = 0.034, and η^2^ = 0.110] indicated that LPC amplitudes elicited by S1 were higher after (181.41 ± 37.09 μV × ms) than before tDCS (135.60 ± 35.34 μV × ms; and *p* = 0.006), while no pre-post differences were observed in SHAM (*p* = 0.993). No pre-post differences were observed on LPC amplitudes elicited by S2 in tDCS or SHAM groups (all ps ≥ 0.196).

The additional MANOVAs on LPC amplitudes for each ROI yielded significant effects only at *frontal* and/or *central* electrode locations. Thus, significant effects were observed due to *time* [*frontal*: *F*(1,39) = 11.82, *p* = 0.001, and η^2^ = 0.233], *stimulus* × *time* [*frontal*: *F*(1,39) = 5.74, *p* = 0.022, and η^2^ = 0.128; *central*: *F*(1,39) = 7.12, *p* = 0.011, and η^2^ = 0.154], *hemisphere* × *group* [*central*: *F*(1,39) = 4.56, *p* = 0.039, and η^2^ = 0.105] and *stimulus* × *time* × *group* [*frontal*: *F*(1,39) = 10.64, *p* = 0.002, and η^2^ = 0.214; *central*: *F*(1,39) = 5.10, *p* = 0.030, and η^2^ = 0.116]. *Post hoc* mean comparisons further revealed that LPC amplitudes elicited by S1 were higher after than before tDCS at *frontal* (*p* ≤ 0.001) and *central* (*p* = 0.009).

### Effects of Anodal tDCS on Sensory Gating of P50, N100, and LPC Amplitudes

**FIGURE 3:**
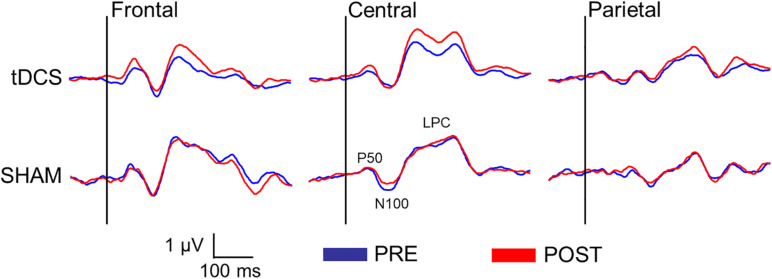
Difference waveforms (S1 minus S2) before and after the intervention in both groups (tDCS vs. SHAM) at three regions of interest (ROI) (frontal: Fz, F3, F4; central: Cz, C3, C4; and parietal: Pz, P3, P4) representing the sensory gating of the somatosensory evoked potentials (SEPs).

Figure 3 displays the difference waveforms elicited by S1 minus S2, before and after intervention for both groups (tDCS vs. SHAM) at the three ROIs (frontal, central, and parietal). Table 3 displays mean and standard deviations of the sensory gating scores for all SEP components.

**TABLE 3:** Mean (±SD) of the sensory gating scores (S1 minus S2) in the tDCS and SHAM group, before and after stimulation.

Time	Component (peak/area)	ROI	Sensory gating score	
			tDCS	SHAM
Before stimulation	P50 (μV)	Frontal	0.34 (0.53)	0.40 (0.72)
		Central	0.45 (0.69)	0.38 (0.49)
		Parietal	0.05 (0.48)	0.25 (0.47)
	N100 (μV)	Frontal	−0.11 (0.85)	−0.38 (1.18)
		Central	−0.19 (0.46)	−0.56 (0.98)
		Parietal	−0.06 (0.73)	0.02 (0.78)
	LPC (μV × ms)	Frontal	49.31 (92.43)	225.65 (212.75)
		Central	229.30 (425.61)	217.15 (271.61)
		Parietal	103.82 (155.31)	27.97 (127.08)
After stimulation	P50 (μV)	Frontal	0.58 (1.21)	0.36 (0.67)
		Central	0.59 (1.13)	0.46 (0.61)
		Parietal	0.01 (1.07)	0.28 (0.42)
	N100 (μV)	Frontal	0.10 (1.19)	−0.24 (1.41)
		Central	−0.33 (0.62)	−0.34 (0.71)
		Parietal	0.05 (1.09)	0.03 (0.98)
	LPC (μV × ms)	Frontal	115.41 (161.07)	215.38 (222.26)
		Central	311.44 (489.25)	221.66 (246.89)
		Parietal	131.54 (233.46)	33.49 (168.96)

*ROI, regions of interest.*

#### P50 Sensory Gating

The ANOVA revealed a significant main effect of *ROI* [*F*(2, 38) = 3.99, *p* = 0.042, and η^2^ = 0.093]. However, *post hoc* mean comparison analyses did not reveal significant differences between ROIs on sensory gating scores regarding the P50 amplitudes (all ps ≥ 0.079). Moreover, no significant effects due to *time*, group or *time* × *group* were observed on P50 amplitudes at any of the three ROIs.

#### N100 Sensory Gating

No significant effects were observed on sensory gating of N100 amplitudes.

#### LPC Gating

Significant effects due to *ROI* [*F*(2, 38) = 5.77, *p* = 0.006, and η^2^ = 0.129], *time* (pre vs. post) [*F*(1, 39) = 5.01, *p* = 0.031, and η^2^ = 0.114], and *time* × *group* [*F*(1, 39) = 5.04, *p* = 0.031, and η^2^ = 0.114] were found. *Post hoc* mean comparisons of the *time* × *group* effect indicated that sensory gating was more increased after (186.13 ± 41.16 μV × ms) than before tDCS (127.48 ± 36.28 μV × ms; *p* = 0.003), whereas no significant difference was observed in the SHAM group (*p* = 0.997). Finally, separate MANOVAs at each ROI revealed that the significant effects due to *time*, *group* and *time* × *group* appeared only over *frontal* and/or *central* regions: *time* [*central*: *F*(1,39) = 6.50, *p* = 0.015, and η^2^ = 0.143], *group* [*frontal*: *F*(1,39) = 6.69, *p* = 0.014, and η^2^ = 0.146] and *time* × *group* were found [*frontal*: *F*(1,39) = 5.59, *p* = 0.023, and η^2^ = 0.125; *central*: *F*(1,39) = 5.22, *p* = 0.028, and η^2^ = 0.118]. Again, *post hoc* mean comparisons indicated that sensory gating was significantly augmented after than before tDCS at *frontal* (*p* = 0.009) and *central* electrode locations (*p* = 0.006).

## Discussion

In the present study, we examined the effects of anodal tDCS over the somatosensory cortex on brain processing of repetitive tactile stimuli in healthy individuals. For this purpose, SEPs elicited by the tactile stimulation of the dominant hand were recorded by using a S1/S2 paired stimulus paradigm before and after tDCS was applied over the contralateral somatosensory cortex (CP3 or CP4 electrode location). Consistent with previous studies using electrical, laser or vibrotactile stimulation (Dieckhöfer et al., 2006; Antal et al., 2008; Lenoir et al., 2017), we found that anodal tDCS was able to modulate brain excitability by enhancing SEP amplitudes to non-painful tactile stimuli. In the present study, anodal tDCS led to increased sensory gating of the late positive complex (LPC; 150–350 ms after stimulus onset) at frontal and central regions, whereas sham stimulation did not produce any changes in sensory gating. Our results further suggested that this sensory gating enhancement could be due to augmented LPC amplitudes in response to S1 elicited by tDCS intervention.

Sensory gating is an inhibitory mechanism of the central nervous system to reduce brain processing of repetitive and redundant sensory information (Freedman et al., 1987; Cromwell et al., 2008). This phenomenon is usually observed in the auditory system by using paired-pulse stimulation tasks, in which two identical stimuli are successively presented. It has been suggested that the first stimulus of each S1/S2 pair could activate some inhibitory brain pathways that suppress or attenuate the response to the second stimulus presented a short time later (e.g., 500 ms; Boutros and Belger, 1999). Indeed, animal research has demonstrated that the first stimulus in such pulse-paired stimulation tasks activates excitatory pyramidal cells, as well as inhibitory hippocampal interneurons, that would suppress the activity in the pyramidal neurons elicited by subsequent presentations of the second stimulus (Miller and Freedman, 1995). Neuropharmacological research has further revealed that cholinergic, dopaminergic, and noradrenergic neurotransmitter systems are involved in the modulation of brain responses to repetitive stimulation (Leonard et al., 1996). Furthermore, this inhibitory mechanism toward repetitive stimuli has been linked to different cognitive functions underlying information processing such as discriminability, response bias, behavioral inhibition, working memory, and attention (Lijffijt et al., 2009), and it has been suggested that widespread attentional cerebral networks, including frontal and inferior parietal lobes, dorsal anterior cingulate, thalamus, and brainstem could be involved (Sturm and Willmes, 2001; Fan et al., 2005, 2007; Kellermann et al., 2011). Our findings of lower amplitudes of early- (P50) and middle-latency components (N100 and LPC) of SEPs elicited by S2 after presenting S1 are in agreement with previous studies examining sensory gating in the auditory (Johnson and Adler, 1993; Lijffijt et al., 2009) and other somatosensory modalities such as the electrical stimulation to the median nerve (Stevenson et al., 2012; Cheng et al., 2016, 2017; Panther et al., 2019; Takeuchi et al., 2019), thus confirming that our repetitive tactile paired-pulse stimulation task was appropriate to elicit this central inhibitory mechanism (Montoya et al., 2006; Terrasa et al., 2018). In the present study, the fact that the amplitude reductions elicited by the second stimulus with respect to the first stimulus appeared in all three SEP components (P50, N100, and LPC) further reveals that this inhibitory mechanism recruit widespread regions of the brain (Zhang et al., 2011; Terrasa et al., 2018). Indeed, previous studies have shown that somatosensory P50 responses represent the primary evoked cortical response to tactile stimulation (Hari et al., 1984), whereas N100 amplitudes seem to be generated in the secondary somatosensory cortex, distributed mostly over fronto-central regions and modulated by attentional processes (Näätänen and Picton, 1987). Moreover, LPC amplitudes have been associated with more complex cognitive functioning, such as orienting, stimulus evaluation, memory, and decision making (Pritchard, 1981; Johnson, 1986; Polich and Herbst, 2000), and are generated in precentral and postcentral gyri, anterior cingulate cortex and insula during paired-pulse stimulation tasks (Annic et al., 2016; Terrasa et al., 2018). To the best our knowledge, this is the first study demonstrating tDCS modulation effects in both early and late SEP components compared with previous studies only observing modulatory effects restricted to early components.

More interestingly, our findings demonstrated that anodal tDCS of the somatosensory cortex may lead to a significant improvement of the inhibitory mechanism associated with repetitive and redundant somatosensory information processing in healthy individuals. Thus, we observed that the sensory gating response (S1 minus S2) increased after tDCS, but not after SHAM. Nevertheless, this modulatory effect was statistically significant only for LPC amplitudes at frontal and central electrodes, indicating that anodal tDCS caused a stronger attenuation of brain responses to redundant stimuli by improving cognitive processes such as attention, evaluation, decision making, and orientation toward somatosensory information. In agreement with these findings, previous research has shown that anodal tDCS may enhance tactile spatial acuity (Ragert et al., 2008; Fujimoto et al., 2014, 2016), as well as visuospatial attention and alerting (Sparing et al., 2009; Bolognini et al., 2010; Coffman et al., 2012). Thus, our data appear to confirm that the enhancement of cortical excitability caused by anodal tDCS would also result in an improvement of brain processing linked to cognitive processes such as attention and evaluation of bodily information. Moreover, considering that source generators of LPC amplitudes during a paired-pulse stimulation task seem to be located within precentral and postcentral gyri, anterior cingulate cortex and insula (Annic et al., 2016; Terrasa et al., 2018), it could be that anodal tDCS was specifically activating some neural mechanism to improve cognitive factors involved in such inhibitory processes. Further research examining the modulatory effects of tDCS on sensory gating should address the question whether tDCS can differentially affect sensory/perceptual and cognitive aspects of information brain processing during sensory gating.

Another important finding of the present study was that sensory gating of the P50 and N100 amplitudes was not affected by tDCS, meaning that the second stimulus of the paired-pulse stimulation task was attenuated at these early latencies of the SEPs in a similar way before and after anodal tDCS. Considering that previous research has shown that anodal tDCS produces a significant amplitude enhancement of early SEP components elicited by electrical stimulation (Matsunaga et al., 2004), the lack of modulatory effects in sensory gating of P50 and N100 amplitudes could be due to the fact that anodal tDCS was producing a general and unspecific effect over the cortex without affecting the input registration at the early stages of information processing. Consistent with this interpretation, a recent study examining the effects of tDCS over the dorsolateral prefrontal cortex on auditory sensory gating found a significant reduction of sensory gating after cathodal tDCS but was unable to find significant effects on P50 after anodal tDCS (Terada et al., 2015). The authors suggested that cathodal tDCS might have weakened the habitual inhibitory effect of the dorsolateral prefrontal cortex on sensory gating, contributing to the reduction of the P50 amplitude elicited by S1, and therefore leading to a deficit in sensory gating. Conversely, the anodal tDCS of the somatosensory cortex in the present study could have strengthened the cortical excitability and led to an enhancement of P50 amplitudes elicited by both S1 and S2, which in turn would have produced no change in sensory gating. Further research on the differential modulatory effects of cathodal and anodal tDCS of the dorsolateral prefrontal and somatosensory cortices, and its effects on somatosensory processing and somatosensory gating would be an intriguing proposition.

Some limitations of the present study should be considered. First, the results of the study might be biased by the small sample size and the inter-subject design. Second, tDCS is considered to cause widespread modulation of the brain and its effects depend on the basal state of the brain at the time of application (Woods et al., 2016). Although we have tried to keep the brain state of all participants at the same level by introducing an initial adaptation period, the influence of the individualś thoughts, beliefs, or expectations before the treatment could not be controlled. Perhaps a within-subject (crossover) design in which participants are randomized to receive both active and simulated sessions with a time interval, could help to minimize these effects. Third, considering that neuromodulatory effects may be better accomplished by pairing electrical brain stimulation with the behavior to be modulated (Woods et al., 2016), a closer presentation of transcranial electrical stimulation and tactile stimuli, as it occurs during the application of tACS, would help for a better understanding of the neurophysiological effects of neuromodulation on somatosensory processing. In the same vein, the inclusion of an additional control condition using a different stimulation site and a more detailed analysis of changes in somatosensory processing during the application of tDCS would be useful in future research. In the present study, such analysis could not be carried out due to the large number of artifacts that were observed during the simultaneous recording of the EEG and the application of tDCS. Fourth, the fact that changes on sensory gating may be due to S1 enhancement should not be disregard. Finally, our discussion about brain structures that could be affected by tDCS was based on previous research, and a combined EEG recording with functional magnetic resonance imaging would have shed more light on the neurophysiological changes associated with tDCS during somatosensory processing.

## Conclusion

In conclusion, our results suggest that somatosensory gating elicited by the paired-pulse presentation of non-painful tactile stimuli could be modulated by anodal tDCS over the somatosensory cortex. A greater suppression of LPC amplitudes (time window 150–350 ms after stimulus onset) of the SEPs was observed at frontal and central regions after anodal tDCS, whereas no effects were observed in the attenuation of P50 and N100 amplitudes. Results suggest that anodal tDCS may influence the inhibitory brain mechanism involved in the processing of bodily information by enhancing cortical excitability and strengthening the cognitive and neurophysiological mechanisms involved in the suppression of repetitive and redundant information.

## Data Availability Statement

The raw data supporting the conclusion of this article will be made available by the authors, without undue reservation.

## Ethics Statement

The studies involving human participants were reviewed and approved by Ethics Committee of the Balearic Islands (Spain). The patients/participants provided their written informed consent to participate in this study.

## Author Contributions

CM and PM contributed significantly to the design of the study and wrote most of the manuscript. CM and CW collected the data. CM and JT performed the data analyses. PM critically revised the manuscript. All authors contributed to the article and approved the submitted version.

## Conflict of Interest

The authors declare that the research was conducted in the absence of any commercial or financial relationships that could be construed as a potential conflict of interest.

## Publisher’s Note

All claims expressed in this article are solely those of the authors and do not necessarily represent those of their affiliated organizations, or those of the publisher, the editors and the reviewers. Any product that may be evaluated in this article, or claim that may be made by its manufacturer, is not guaranteed or endorsed by the publisher.

## Footnotes

1
www.neurobs.com

## References

[B1] Ahn H. Woods A. J. Kunik M. E. Bhattacharjee A. Chen Z. Choi E. (2017). Efficacy of transcranial direct current stimulation over primary motor cortex (anode) and contralateral supraorbital area (cathode) on clinical pain severity and mobility performance in persons with knee osteoarthritis: an experimenter- and participant-blinded, randomized, sham-controlled pilot clinical study. *Brain Stimul.* 10 902–909. 10.1016/j.brs.2017.05.007 28566193 PMC5568498

[B2] Angius L. Santarnecchi E. Pascual-Leone A. Marcora S. M. (2019). Transcranial Direct Current Stimulation over the Left Dorsolateral Prefrontal Cortex Improves Inhibitory Control and Endurance Performance in Healthy Individuals. *Neuroscience* 419 34–45. 10.1016/j.neuroscience.2019.08.052 31493549

[B3] Annic A. Bourriez J. Delval A. Bocquillon P. Trubert C. Derambure P. (2016). Effects of Stimulus-Driven and Goal-Directed Attention on Prepulse Inhibition of Brain Oscillations. *Front. Hum. Neurosci.* 10:390. 10.3389/fnhum.2016.00390 27524966 PMC4965466

[B4] Antal A. Alekseichuk I. Bikson M. Brockmöller J. Brunoni A. R. Chen R. (2017). Low intensity transcranial electric stimulation: safety, ethical, legal regulatory and application guidelines. *Clin. Neurophysiol.* 128 1774–1809. 10.1016/j.clinph.2017.06.001 28709880 PMC5985830

[B5] Antal A. Brepohl N. Poreisz C. Boros K. Csifcsak G. Paulus W. (2008). Transcranial direct current stimulation over somatosensory cortex decreases experimentally Induced acute pain perception. *Clin. J. Pain* 24 56–63. 10.1097/AJP.0b013e318157233b 18180638

[B6] Antal A. Nitsche M. A. Paulus W. (2006). Transcranial direct current stimulation and the visual cortex. *Brain Res. Bull.* 68 459–463. 10.1016/j.brainresbull.2005.10.006 16459203

[B7] Antal A. Polania R. Schmidt-Samoa C. Dechent P. Paulus W. (2011). Transcranial direct current stimulation over the primary motor cortex during fMRI. *Neuroimage* 55 590–596. 10.1016/j.neuroimage.2010.11.085 21211569

[B8] Bagby R. M. Parker J. D. A. Taylor G. J. (1994). The twenty-item Toronto Alexithymia scale-I. Item selection and cross-validation of the factor structure. *J. Psychosom. Res.* 38 23–32. 10.1016/0022-3999(94)90005-1 8126686

[B9] Beck A. T. Ward C. H. Mendelson M. Mock J. Erbaugh J. (1961). An Inventory for Measuring Depression. *Arch. Gen. Psychiatry* 4 561–571. 10.1001/archpsyc.1961.01710120031004 13688369

[B10] Bolognini N. Olgiati E. Rossetti A. Maravita A. (2010). Enhancing multisensory spatial orienting by brain polarization of the parietal cortex. *Eur. J. Neurosci.* 31 1800–1806. 10.1111/j.1460-9568.2010.07211.x 20584184

[B11] Boutros N. N. Belger A. (1999). Midlatency evoked potentials attenuation and augmentation reflect different aspects of sensory gating. *Biol. Psychiatry* 45 917–922. 10.1016/s0006-3223(98)00253-4 10202580

[B12] Cheng C. H. Chan P. Y. S. Niddam D. M. Tsai S. Y. Hsu S. C. Liu C. Y. (2016). Sensory gating, inhibition control and gamma oscillations in the human somatosensory cortex. *Sci. Rep.* 6:20437. 10.1038/srep20437 26843358 PMC4740805

[B13] Cheng C. H. Tsai S. Y. Liu C. Y. Niddam D. M. (2017). Automatic inhibitory function in the human somatosensory and motor cortices: an MEG-MRS study. *Sci. Rep.* 7:4234. 10.1038/s41598-017-04564-1 28652623 PMC5484662

[B14] Chrysikou E. G. Wing E. K. van Dam W. O. (2019). Transcranial direct current stimulation over the prefrontal cortex in depression modulates cortical excitability in emotion regulation regions as measured by concurrent functional magnetic resonance imaging: an exploratory study. *Biol. Psychiatry Cogn. Neurosci. Neuroimag*. 10.1016/j.bpsc.2019.12.004 32111579

[B15] Coffman B. A. Trumbo M. C. Clark V. P. (2012). Enhancement of object detection with transcranial direct current stimulation is associated with increased attention. *BMC Neurosci.* 13:108. 10.1186/1471-2202-13-108 22963503 PMC3494452

[B16] Cromwell H. C. Mears R. P. Wan L. Boutros N. N. (2008). Sensory gating: a translational effort from basic to clinical science. *Clin. EEG Neurosci.* 39 69–72. 10.1177/155005940803900209 18450171 PMC4127047

[B17] Dieckhöfer A. Waberski T. D. Nitsche M. Paulus W. Buchner H. Gobbelé R. (2006). Transcranial direct current stimulation applied over the somatosensory cortex - Differential effect on low and high frequency SEPs. *Clin. Neurophysiol.* 117 2221–2227. 10.1016/j.clinph.2006.07.136 16931142

[B18] Fan J. Byrne J. Worden M. S. Guise K. G. McCandliss B. D. Fossella J. (2007). The relation of brain oscillations to attentional networks. *J. Neurosci.* 27 6197–6206. 10.1523/JNEUROSCI.1833-07.2007 17553991 PMC6672149

[B19] Fan J. McCandliss B. D. Fossella J. Flombaum J. I. Posner M. I. (2005). The activation of attentional networks. *Neuroimage* 26 471–479. 10.1016/j.neuroimage.2005.02.004 15907304

[B20] Freedman R. Adler L. E. Gerhardt G. A. Waldo M. Baker N. Rose G. M. (1987). Neurobiological studies of sensory gating in schizophrenia. *Schizophr. Bull.* 13 669–678. 10.1093/schbul/13.4.669 2894074

[B21] Fregni F. Boggio P. S. Nitsche M. Bermpohl F. Antal A. Feredoes E. (2005). Anodal transcranial direct current stimulation of prefrontal cortex enhances working memory. *Exp. Brain Res.* 166 23–30. 10.1007/s00221-005-2334-6 15999258

[B22] Fregni F. Orsati F. Pedrosa W. Fecteau S. Tome F. A. M. Nitsche M. A. (2008). Transcranial direct current stimulation of the prefrontal cortex modulates the desire for specific foods. *Appetite* 51 34–41. 10.1016/j.appet.2007.09.016 18243412 PMC3541023

[B23] Fuerst D. R. Gallinat J. Boutros N. N. (2007). Range of sensory gating values and test-retest reliability in normal subjects. *Psychophysiology* 44 620–626. 10.1111/j.1469-8986.2007.00524.x 17437554

[B24] Fujimoto S. Kon N. Otaka Y. Yamaguchi T. Nakayama T. Kondo K. (2016). Transcranial Direct Current Stimulation Over the Primary and Secondary Somatosensory Cortices Transiently Improves Tactile Spatial Discrimination in Stroke Patients. *Front. Neurosci.* 10:128. 10.3389/fnins.2016.00128 27064531 PMC4814559

[B25] Fujimoto S. Yamaguchi T. Otaka Y. Kondo K. Tanaka S. (2014). Dual-hemisphere transcranial direct current stimulation improves performance in a tactile spatial discrimination task. *Clin. Neurophysiol.* 125 1669–1674. 10.1016/j.clinph.2013.12.100 24411524

[B26] Furubayashi T. Terao Y. Arai N. Okabe S. Mochizuki H. Hanajima R. (2008). Short and long duration transcranial direct current stimulation (tDCS) over the human hand motor area. *Exp. Brain Res.* 185 279–286. 10.1007/s00221-007-1149-z 17940759

[B27] Gallucci A. Riva P. Romero Lauro L. J. Bushman B. J. (2020). Stimulating the ventrolateral prefrontal cortex (VLPFC) modulates frustration-induced aggression: a tDCS experiment. *Brain Stimul.* 13 302–309. 10.1016/j.brs.2019.10.015 31676301

[B28] Gibson S. J. Littlejohn G. O. Gorman M. M. Helme R. D. Granges G. (1994). Altered heat pain thresholds and cerebral event-related potentials following painful CO2 laser stimulation in subjects with fibromyalgia syndrome. *Pain* 58 185–193. 10.1016/0304-3959(94)90198-8 7816486

[B29] Gratton G. Coles M. G. Donchin E. (1983). A new method for off-line removal of ocular artifact. *Electroencephalogr. Clin. Neurophysiol.* 55 468–484. 10.1016/0013-4694(83)90135-9 6187540

[B30] Grundmann L. Rolke R. Nitsche M. A. Pavlakovic G. Happe S. Treede R. D. (2011). Effects of transcranial direct current stimulation of the primary sensory cortex on somatosensory perception. *Brain Stimul.* 4 253–260. 10.1016/j.brs.2010.12.002 22032740

[B31] Hari R. Reinikainen K. Kaukoranta E. Hämäläinen M. Ilmoniemi R. Penttinen A. (1984). Somatosensory evoked cerebral magnetic fields from SI and SII in man. *Electroencephalogr. Clin. Neurophysiol.* 57 254–263. 10.1016/0013-4694(84)90126-3 6199186

[B32] Johnson M. R. Adler L. E. (1993). Transient impairment in P50 auditory sensory gating induced by a cold-pressor test. *Biol. Psychiatry* 33 380–387. 10.1016/0006-3223(93)90328-B 8386006

[B33] Johnson R. (1986). A triarchic model of P300 amplitude. *Psychophysiology* 23 367–384. 10.1111/j.1469-8986.1986.tb00649.x 3774922

[B34] Kellermann T. Reske M. Jansen A. Satrapi P. Shah N. J. Schneider F. (2011). Latencies in BOLD response during visual attention processes. *Brain Res.* 1386 127–138. 10.1016/j.brainres.2011.02.023 21329677

[B35] Lefaucheur J.-P. (2008). Principles of therapeutic use of transcranial and epidural cortical stimulation. *Clin. Neurophysiol.* 119 2179–2184. 10.1016/j.clinph.2008.07.007 18762449

[B36] Lefaucheur J. P. Antal A. Ayache S. S. Benninger D. H. Brunelin J. Cogiamanian F. (2017). Evidence-based guidelines on the therapeutic use of transcranial direct current stimulation (tDCS). *Clin. Neurophysiol.* 128 56–92. 10.1016/j.clinph.2016.10.087 27866120

[B37] Lenoir C. Huang G. Vandermeeren Y. Hatem S. M. Mouraux A. (2017). Human primary somatosensory cortex is differentially involved in vibrotaction and nociception. *J. Neurophysiol.* 118 317–330. 10.1152/jn.00615.2016 28446584 PMC5498736

[B38] Leonard S. Adams C. Breese C. R. Adler L. E. Bickford P. Byerley W. (1996). Nicotinic Receptor Function in Schizophrenia. *Schizophr. Bull.* 22 431–446. 10.1093/schbul/22.3.431 8873294

[B39] Lijffijt M. Lane S. D. Meier S. L. Boutros N. N. Burroughs S. Steinberg J. L. (2009). P50, N100, and P200 sensory gating: relationships with behavioral inhibition, attention, and working memory. *Psychophysiology* 46 1059–1068. 10.1111/j.1469-8986.2009.00845.x 19515106 PMC2821570

[B40] Lorenz J. Grasedyck K. Bromm B. (1996). Middle and long latency somatosensory evoked potentials after painful laser stimulation in patients with fibromyalgia syndrome. *Electroencephalogr. Clin. Neurophysiol.* 100 165–168. 10.1016/0013-4694(95)00259-6 8617155

[B41] Martínez-Jauand M. González-Roldán A. M. A. M. Muñoz M. A. M. A. Sitges C. Cifre I. Montoya P. (2012). Somatosensory activity modulation during observation of other’s pain and touch. *Brain Res.* 1467 48–55. 10.1016/j.brainres.2012.05.055 22683688

[B42] Matsumoto H. Ugawa Y. (2017). Adverse events of tDCS and tACS: a review. *Clin. Neurophysiol. Pract.* 2 19–25. 10.1016/j.cnp.2016.12.003 30214966 PMC6123849

[B43] Matsunaga K. Nitsche M. A. Tsuji S. Rothwell J. C. (2004). Effect of transcranial DC sensorimotor cortex stimulation on somatosensory evoked potentials in humans. *Clin. Neurophysiol.* 115 456–460. 10.1016/S1388-2457(03)00362-6 14744588

[B44] Miller C. L. Freedman R. (1995). The activity of hippocampal interneurons and pyramidal cells during the response of the hippocampus to repeated auditory stimuli. *Neuroscience* 69 371–381. 10.1016/0306-4522(95)00249-i 8552235

[B45] Moliadze V. Atalay D. Antal A. Paulus W. (2012). Close to threshold transcranial electrical stimulation preferentially activates inhibitory networks before switching to excitation with higher intensities. *Brain Stimul.* 5 505–511. 10.1016/j.brs.2011.11.004 22445135

[B46] Montoya P. Pauli P. Batra A. Wiedemann G. (2005). Altered processing of pain-related information in patients with fibromyalgia. *Eur. J. Pain* 9 293–303. 10.1016/j.ejpain.2004.07.012 15862479

[B47] Montoya P. Sitges C. García-Herrera M. Rodríguez-Cotes A. Izquierdo R. Truyols M. (2006). Reduced brain habituation to somatosensory stimulation in patients with fibromyalgia. *Arthritis Rheum.* 54 1995–2003. 10.1002/art.21910 16732548

[B48] Näätänen R. Picton T. (1987). The N1 wave of the human electric and magnetic response to sound: a review and an analysis of the component structure. *Psychophysiology* 24 375–425. 10.1111/j.1469-8986.1987.tb00311.x 3615753

[B49] Nitsche M. A. Cohen L. G. Wassermann E. M. Priori A. Lang N. Antal A. (2008). Transcranial direct current stimulation: state of the art 2008. *Brain Stimul.* 1 206–223. 10.1016/j.brs.2008.06.004 20633386

[B50] Nitsche M. A. Paulus W. (2000). Excitability changes induced in the human motor cortex by weak transcranial direct current stimulation. *J. Physiol.* 527 633–639. 10.1111/j.1469-7793.2000.t01-1-00633.x 10990547 PMC2270099

[B51] Nitsche M. A. Paulus W. (2001). Sustained excitability elevations induced by transcranial DC motor cortex stimulation in humans. *Neurology* 57 1899–1901. 10.1212/WNL.57.10.1899 11723286

[B52] Nitsche M. A. Schauenburg A. Lang N. Liebetanz D. Exner C. Paulus W. (2003). Facilitation of implicit motor learning by weak transcranial direct current stimulation of the primary motor cortex in the human. *J. Cogn. Neurosci.* 15 619–626. 10.1162/089892903321662994 12803972

[B53] Oldfield R. C. (1971). The assessment and analysis of handedness: the Edinburgh inventory. *Neuropsychologia* 9 97–113. 10.1016/0028-3932(71)90067-4 5146491

[B54] Panther P. Kuehne M. Voges J. Nullmeier S. Kaufmann J. Hausmann J. (2019). Electric stimulation of the medial forebrain bundle influences sensorimotor gaiting in humans. *BMC Neurosci.* 20:20. 10.1186/s12868-019-0503-y 31035935 PMC6489177

[B55] Paulus W. (2011). Transcranial electrical stimulation (tES - tDCS; tRNS, tACS) methods. *Neuropsychol. Rehabil.* 21 602–617. 10.1080/09602011.2011.557292 21819181

[B56] Polich J. Herbst K. L. (2000). P300 as a clinical assay: rationale, evaluation, and findings. *Int. J. Psychophysiol.* 38 3–19. 10.1016/S0167-8760(00)00127-6 11027791

[B57] Pritchard W. S. (1981). Psychophysiology of P300. *Psychol. Bull.* 89 506–540. 10.1037/0033-2909.89.3.506 7255627

[B58] Ragert P. Vandermeeren Y. Camus M. Cohen L. G. (2008). Improvement of spatial tactile acuity by transcranial direct current stimulation. *Clin. Neurophysiol.* 119 805–811. 10.1016/j.clinph.2007.12.001 18203660 PMC2394720

[B59] Saito K. Otsuru N. Inukai Y. Miyaguchi S. Yokota H. Kojima S. (2019). Comparison of transcranial electrical stimulation regimens for effects on inhibitory circuit activity in primary somatosensory cortex and tactile spatial discrimination performance. *Behav. Brain Res.* 375:112168. 10.1016/j.bbr.2019.112168 31442547

[B60] Shilo G. Lavidor M. (2019). Non-linear effects of cathodal transcranial direct current stimulation (tDCS) of the primary motor cortex on implicit motor learning. *Exp. Brain Res.* 237 919–925. 10.1007/s00221-019-05477-3 30661087

[B61] Sparing R. Thimm M. Hesse M. D. Küst J. Karbe H. Fink G. R. (2009). Bidirectional alterations of interhemispheric parietal balance by non-invasive cortical stimulation. *Brain* 132 3011–3020. 10.1093/brain/awp154 19528092

[B62] Spielberger C. D. Gorsurch R. L. Lushene R. E. (1970). *The State-Trait Anxiety Inventory (STAI): Test Manual.* Palo Alto: Consulting Psychologists Press.

[B63] Stagg C. J. Lin R. L. Mezue M. Segerdahl A. Kong Y. Xie J. (2013). Widespread modulation of cerebral perfusion induced during and after transcranial direct current stimulation applied to the left dorsolateral prefrontal cortex. *J. Neurosci.* 33 11425–11431. 10.1523/JNEUROSCI.3887-12.2013 23843514 PMC3724554

[B64] Stagg C. J. Nitsche M. A. (2011). Physiological basis of transcranial direct current stimulation. *Neuroscientist* 17 37–53. 10.1177/1073858410386614 21343407

[B65] Stevenson C. M. Wang F. Brookes M. J. Zumer J. M. Francis S. T. Morris P. G. (2012). Paired pulse depression in the somatosensory cortex: associations between MEG and BOLD fMRI. *Neuroimage* 59 2722–2732. 10.1016/j.neuroimage.2011.10.037 22036680

[B66] Sturm W. Willmes K. (2001). On the functional neuroanatomy of intrinsic and phasic alertness. *Neuroimage* 14 76–84. 10.1006/nimg.2001.0839 11373136

[B67] Takeuchi N. Kinukawa T. Sugiyama S. Inui K. Kanemoto K. Nishihara M. (2019). Suppression of Somatosensory Evoked Cortical Responses by Noxious Stimuli. *Brain Topogr.* 32 783–793. 10.1007/s10548-019-00721-z 31218521 PMC6707979

[B68] Terada H. Kurayama T. Nakazawa K. Matsuzawa D. Shimizu E. (2015). Transcranial direct current stimulation (tDCS) onthe dorsolateral prefrontal cortex alters P50 gating. *Neurosci. Lett.* 602 139–144. 10.1016/j.neulet.2015.07.003 26163464

[B69] Terrasa J. L. Montoya P. González-Roldán A. M. Sitges C. (2018). Inhibitory Control Impairment on Somatosensory Gating Due to Aging: an Event-Related Potential Study. *Front. Hum. Neurosci.* 12:280. 10.3389/fnhum.2018.00280 30050421 PMC6052091

[B70] Thair H. Holloway A. L. Newport R. Smith A. D. (2017). Transcranial direct current stimulation (tDCS): a Beginner’s guide for design and implementation. *Front. Neurosci.* 11:641. 10.3389/fnins.2017.00641 29213226 PMC5702643

[B71] Vanderhasselt M. A. Sanchez A. Josephy H. Baeken C. Brunoni A. R. De Raedt R. (2017). Anodal tDCS over the right dorsolateral prefrontal cortex modulates cognitive processing of emotional information as a function of trait rumination in healthy volunteers. *Biol. Psychol.* 123 111–118. 10.1016/j.biopsycho.2016.12.006 27979654

[B72] Vaseghi B. Zoghi M. Jaberzadeh S. (2015). How does anodal transcranial direct current stimulation of the pain neuromatrix affect brain excitability and pain perception? A randomised, double-blind, sham-control study. *PLoS One* 10:e0118340. 10.1371/journal.pone.0118340 25738603 PMC4349802

[B73] Woods A. J. Antal A. Bikson M. Boggio P. S. Brunoni A. R. Celnik P. (2016). A technical guide to tDCS, and related non-invasive brain stimulation tools. *Clin. Neurophysiol.* 127 1031–1048. 10.1016/j.clinph.2015.11.012 26652115 PMC4747791

[B74] Zaehle T. Sandmann P. Thorne J. D. Jäncke L. Herrmann C. S. (2011). Transcranial direct current stimulation of the prefrontal cortex modulates working memory performance: combined behavioural and electrophysiological evidence. *BMC Neurosci.* 12:2. 10.1186/1471-2202-12-2 21211016 PMC3024225

[B75] Zaghi S. Acar M. Hultgren B. Boggio P. S. Fregni F. (2010). Noninvasive brain stimulation with low-intensity electrical currents: putative mechanisms of action for direct and alternating current stimulation. *Neuroscientist* 16 285–307. 10.1177/1073858409336227 20040569

[B76] Zhang L. I. Zhou Y. Tao H. W. (2011). Inhibitory synaptic mechanisms underlying functional diversity in auditory cortex. *J. Gen. Physiol.* 138 311–320. 10.1085/jgp.201110650 21875980 PMC3171074

